# The Influence of Li on the Fracture Characteristics and Mechanical Properties of Extruded Beryllium–Aluminum Composites

**DOI:** 10.3390/ma18051055

**Published:** 2025-02-27

**Authors:** Wentong Li, Yixiao Xia, Yutong Sun, Juanrui Hu, Leilei Hao, Yun Liu, Boyu Ju, Guoqin Chen, Wenshu Yang

**Affiliations:** 1 School of Materials Science and Engineering, Harbin Institute of Technology, Harbin 150001, China; hitliwentong@163.com (W.L.); etialxia@icloud.com (Y.X.); sunyt2000@163.com (Y.S.); chenguoqin@hit.edu.cn (G.C.); 2 State Key Laboratory of Precision Welding & Joining of Materials and Structures, Harbin Institute of Technology, Harbin 150001, China; 3 Beijing Institute of Aerospace Control Devices, Beijing 100039, China; 18610697362@163.com (J.H.); 13161880493@163.com (L.H.); 13621067604@163.com (Y.L.); 4 Zhengzhou Research Institute, Harbin Institute of Technology, Zhengzhou 450001, China

**Keywords:** beryllium–aluminum composites, pressure infiltration, Al-Li, mechanical properties

## Abstract

Alloying is an important method to improve the mechanical properties of beryllium–aluminum composites. In this study, two kinds of beryllium–aluminum composites with and without Li were prepared by pressure impregnation method and extrusion, and the effects of Li on the microstructure and mechanical properties of beryllium–aluminum alloy were investigated by XRD, SEM and tensile test. The results show the addition of Li enhances the absorption of oxygen and nitrogen in the alloy; however, there is no significant change in the material’s density, which remains at 2.07 g/cm^3^. Despite an exacerbation of debonding phenomena at the fracture surface of Li-containing beryllium–aluminum alloys and a decrease in ductile dimples density, the yield strength increased from 266.2 MPa to 317.1 MPa, the tensile strength increased from 348.6 MPa to 411.4 MPa, and the elongation only decreased slightly from 2.9% to 2.5%. These experimental results support the design and preparation of high-performance beryllium–aluminum composites.

## 1. Introduction

Beryllium–aluminum composite is a type of Al matrix composite with Be as the reinforcement phase. This material exhibits outstanding properties, including high stiffness, low density, excellent thermal stability, and favorable machinability [1,2,3]. For instance, the AlBeMet series products by Materion Corporation (Mayfield Heights, OH, USA) in the U.S. have a density of 2.10 g/cm^3^ and an elastic modulus of 193 GPa. Currently, beryllium–aluminum composites are widely used in aerospace electronics, optical systems, and satellite technology [4].

Beryllium is a chemically active metal that readily oxidizes to form beryllium oxide (BeO) when exposed to air [5]. BeO between the aluminum and beryllium phases reduces their binding strength. According to the modified shear-lag model, a reduction in the bonding strength between the reinforcement and the matrix adversely affects the transfer of load from the softer matrix material to the harder reinforcement phase. This situation raises the risk of interface cracking, which can harm overall material properties [6]. At present, there has been considerable research on the interface bonding of beryllium–aluminum composites. Kuang et al. prepared Be/Al composites with different BeO content by heating in different gas environments. The findings indicated that the Be/Al composites with a higher BeO content exhibited reduced strength and ductility [7]. In a separate study conducted by the research team, it was discovered that Cu can diffuse from the Al phase to the Be phase. This diffusion process significantly enhances the bonding strength between the Be and Al phases [8]. Xie et al. conducted a first-principles calculation of the beryllium–aluminum composite interface.

The results indicate that the Be(001)/Al(100) interface represents the most stable structural configuration. Furthermore, elements such as Ni, Ag, and Co are found to enhance the bond strength of the Be/Al interface significantly [9]. In the existing studies, improvements in interface bonding strength have mainly been achieved by reducing BeO content or incorporating elements that are soluble in both Be and Al. The BeO between the Be and Al interface is difficult to remove. The oxygen potential of Be is significantly lower than that of Al. This implies that if the Be/Al composite interface is rich in oxygen, the bonding strength between this interface and Al is much weaker than with Be [10]. Lithium with low oxygen potential can be incorporated into the aluminum matrix, potentially enhancing the bonding strength at the interface. This improvement could significantly enhance the mechanical properties of beryllium/aluminum composites.

AlLi alloys are widely utilized in the aerospace industry due to their exceptional stiffness and low density [11,12,13,14]. However, due to the unique chemical properties of Li, the preparation of Al-Li alloy composites presents certain challenges. Currently, the methods available for producing Al-Li matrix composites are relatively limited. By combining Ti powder and B_4_C powder in liquid Al and subjecting the mixture to mechanical stirring, Li et al. developed a composite material featuring an Al-Cu-Li alloy matrix reinforced with TiC+TiB_2_. They investigated the relationship between the T1 precipitated phase and TiC+TiB_2_, as well as examined the precipitation and growth processes of the T1 phase [15]. Wang et al. developed Al-Li matrix composites incorporating B_4_C as the reinforcing particles through the process of accumulative roll bonding(ARB). Through a series of rolling processes, B_4_C particles were uniformly dispersed throughout the material, resulting in simultaneous enhancements in both its plasticity and strength [16]. Ahmad et al. prepared Al-Li-GNP composites through ball milling. The research findings indicated that lithium facilitated the reduction of stacking fault energy, thereby enhancing the ductility of the material. Furthermore, an increase in the fraction of reinforcing particles contributed to the improved strength of the material [17]. However, currently, the research pertaining to the preparation of Al-Li matrix composites through pressure infiltration remains in its nascent stage. The materials produced through pressure infiltration typically exhibit outstanding properties; however, controlling the oxidation reaction during the infiltration process poses significant challenges. It is essential to conduct research on the Al-Li matrix composites that are fabricated through pressure infiltration.

This study prepared beryllium–aluminum composites with an Al-Li matrix for the first time using pressure infiltration. The influence of lithium on the microstructure and mechanical properties of the composite was investigated. The findings of this study serve as a valuable reference for the development of high-performance beryllium–aluminum composites.

## 2. Materials and Methods

### 2.1. Materials and Fabrication

**Figure 1:**
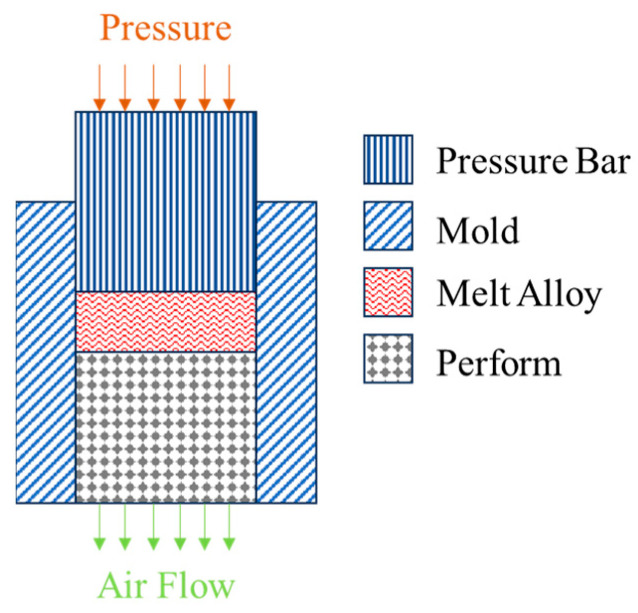
Schematic diagram of the pressure infiltration technology.

Two types of beryllium–aluminum composites were fabricated using pressure infiltration technology, employing pure aluminum and an Al-Li alloy (with a Li content of 0.5 wt.%) as the matrix materials, respectively. The reinforcement utilized in this process was spherical beryllium powder, which was produced through the gas atomization technique. The information on the Be powder can be found in reference [1]. Figure 1 presents a schematic diagram illustrating the pressure infiltration technology. First, beryllium powder is loaded into a steel mold, which is then subjected to pressure to achieve a beryllium fraction of 68 vol.%. This entire process takes place within an argon-filled glove box, and the steel mold features an open top and bottom. Subsequently, the steel mold containing the beryllium powder is preheated at a temperature of 600 °C for two hours. Meanwhile, aluminum ingots were melted at a temperature of 760 °C. Subsequently, the molten pure aluminum (Al-Li alloy) was poured into the preheated mold containing the beryllium preform. Mechanical pressure was then applied to facilitate the infiltration and composite formation process. The gas surrounding the beryllium powders will be expelled from below the mold as pressure is applied. Further details regarding the pressure infiltration technology can be found in the ref. [18]. The composite was extruded at the temperature of 500 °C, with an extrusion ratio of 16:1. Table 1 presents the chemical compositions of the composites.

**Figure 2:**
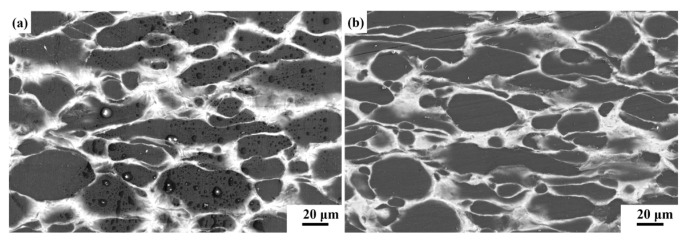
The microstructure of Be/Al (**a**) and Be/AlLi (**b**).

Figure 2 illustrates the microstructure of the two composites, where the Be phase appears dark, and the Al phase appears light. The initial morphology of the Be powder was spherical, while it became elongated following the extrusion process. The resulting composites demonstrate a dense microstructure free of pores.

### 2.2. Characterization

The chemical composition of materials was obtained by inductively coupled plasma optical emission spectrometry (ICP, ICAP7400, Thermo Fisher Scientific, Waltham, MA, USA). The sample was dissolved in the mixture, which was prepared by mixing HCl, HF, and H_2_O in a 1:1:1 ratio. The mixed standard solution of Be, Al, and Li is prepared at a concentration of 100 μg/mL. The specific testing conditions are as follows: argon gas protection, RF power set at 1000 W, plasma flow rate maintained at 15.0 L/min, auxiliary gas flow rate at 1.5 L/min, atomization gas flow rate of 0.6 L/min, observation height fixed at 6 mm, and reading duration established for 5.00 s. The microstructure and fracture morphology of the extruded beryllium–aluminum composites were performed on a scanning electron microscope (Thermo Scientific Scios 2, SEM, Thermo Fisher Scientific, Waltham, MA, USA) equipped with energy dispersive spectroscopy (EDS). The phase compositions of beryllium–aluminum composites were analyzed by X-ray diffraction (XRD, Empyrean, Cu Kα, 15–90°, PANalytical, Almelo, The Netherlands). The Be/AlLi was subjected to analysis using X-ray photoelectron spectroscopy (XPS, ESCLAB 250Xi, Al Kα, Thermo Fisher Scientific, Waltham, MA, USA), and a binary Al-Li alloy was also evaluated for comparative purposes.

**Figure 3:**
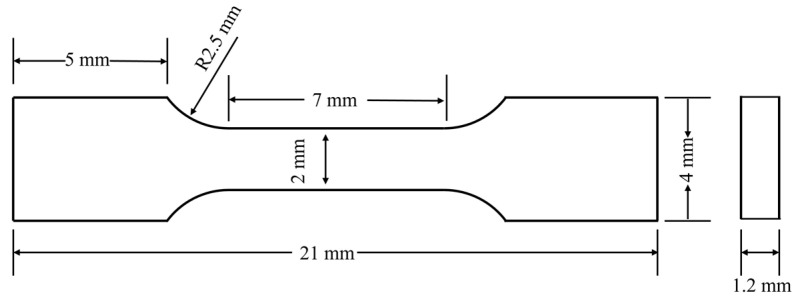
Schematic diagram of the tensile sample sizes.

The tensile properties of beryllium aluminum alloy at room temperature were evaluated using a universal electrical testing machine (Instron 5569, Norwood, MA, USA) with a tensile rate set at 0.5 mm/min. The dimensions of the tensile sample are presented in Figure 3. All tests were conducted more than three times to ensure the reliability and validity of the experimental results. Meanwhile, the density of each group of samples was measured by the Archimedean drainage method, and the results show that both composites exhibit a density of 2.07 g/cm^3^.

## 3. Results

### 3.1. Phase Composition

**Figure 4:**
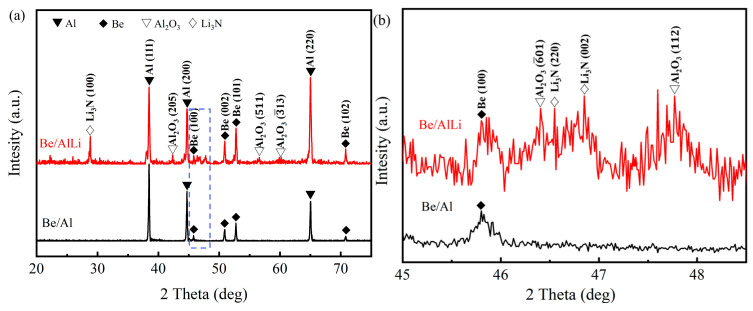
XRD patterns of beryllium–aluminum composites. (**a**) full spectrum; (**b**) the date in the blue box.

The XRD results for Be/Al and Be/AlLi are presented in Figure 4, with Figure 4b providing an enlarged view of the dotted box indicated in Figure 4a. The XRD analysis of Be/Al reveals only the diffraction peaks associated with Be and Al. In contrast, the XRD results for Be/AlLi show relatively prominent peaks corresponding to Li_3_N and Al_2_O_3_. The experimental results align with the chemical properties of Li. During the melting process, an increase in Li content leads to enhanced absorption of hydrogen, nitrogen, and oxygen in liquid aluminum [19]. In addition, the XRD pattern of Be/AlLi exhibits numerous diffraction peaks that are challenging to calibrate, suggesting a highly complex phase composition for Be/AlLi.

**Figure 5:**
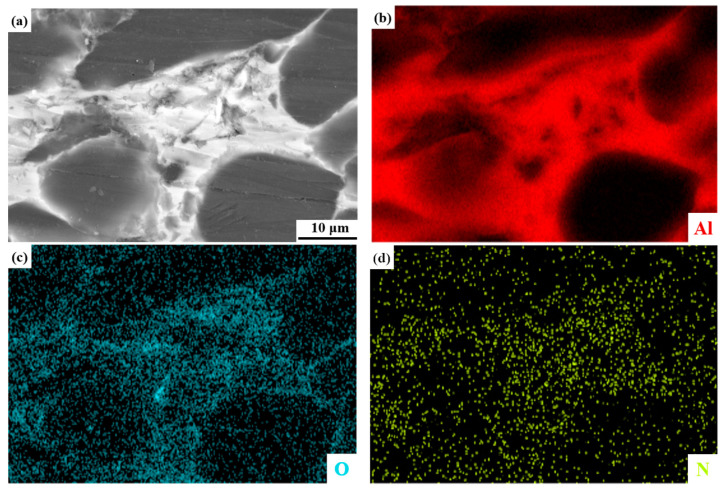
SEM and EDS analysis of Be/AlLi. (**a**) SEM; (**b**–**d**) distribution maps of elements.

To investigate the microstructural characteristics of the Be/AlLi alloy, scanning electron microscopy (SEM) observations and energy dispersive spectroscopy (EDS) analyses were conducted on the microstructure adjacent to the aluminum matrix. The results of these analyses are presented in Figure 5. In Figure 5a, the dark phase represents Be, and the light phase represents Al [20]. In Figure 5c,d, oxygen and nitrogen are concentrated in the Al phase, indicating that oxidation and nitriding processes occurred during the preparation of the Al-Li alloy. This experimental result aligns well with the phase analysis of Be/AlLi presented in Figure 4. On the other hand, the concentration of nitrogen is observed within the aluminum matrix. This indicates that lithium primarily exists in the aluminum phase of the beryllium/aluminum composites, which is also consistent with existing experimental findings [21,22,23,24]. This phenomenon can be attributed to the low solid solubility of both Al and Li in Be [25,26,27], but the solid solubility of Li in the Al phase is relatively high [28].

### 3.2. Mechanical Properties

**Figure 6:**
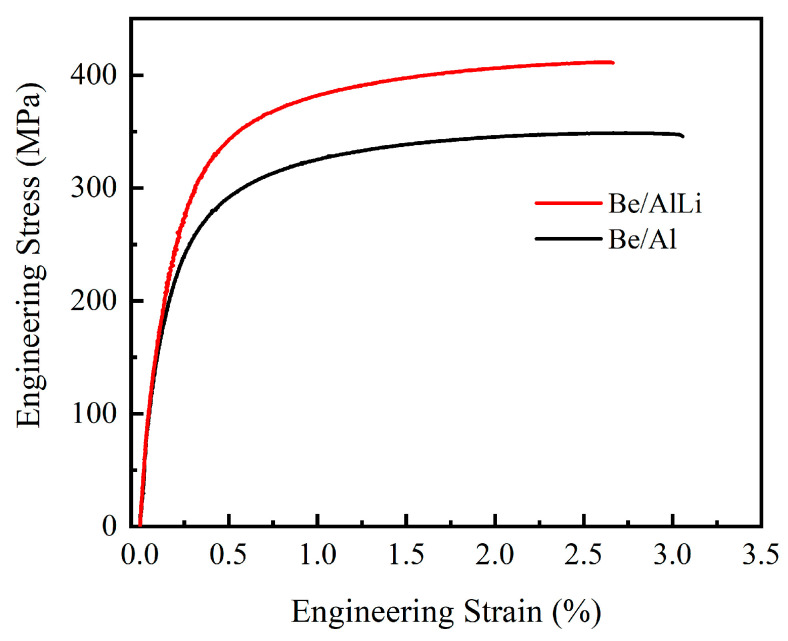
Tensile stress-strain curves of composites.

Some research has indicated that there is minimal difference in the properties measured parallel (RD) and perpendicular (ND) to the extrusion direction of Be/AlLi [18]. Therefore, this study exclusively investigates the mechanical properties of the two composites in the ND direction. Figure 6 presents the tensile stress curves of the two composites. The yield strength (YS) is defined as the stress value corresponding to a strain of 0.2, while the ultimate tensile strength (UTS) represents the maximum stress value observed during testing. The results are summarized in Table 2. The yield strength of Be/Al is measured to be 266.2 ± 6.5 MPa, while the ultimate tensile strength is 348.6 ± 5.7 MPa, and the elongation percentage is found to be 2.9 ± 0.1%. After adding Li, the yield strength and tensile strength of the Be/AlLi alloy exhibit significant improvements, reaching values of 317.1 ± 12.5 MPa and 411.4 ± 3.7 MPa, respectively, which are increased by 50.9 MPa and 62.8 MPa compared with Be/Al. However, the elongation exhibited a slight decrease to 2.5 ± 0.1%.

**Figure 7:**
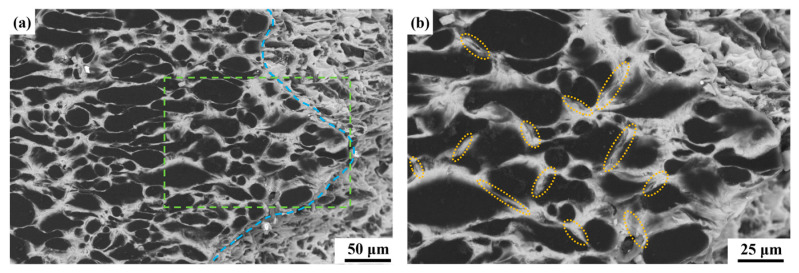

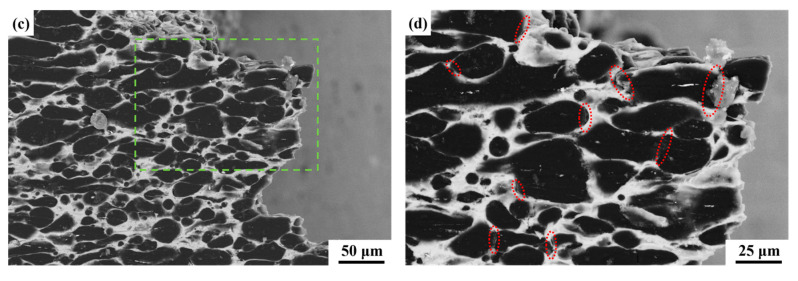
Microstructure of composites in the transverse direction after fracture. (**a**–**b**) Be/Al; (**c**–**d**) Be/AlLi.

In order to investigate the reasons behind the differences in properties between the two composites, an analysis was conducted on the samples following fracture, as shown in Figure 7. Figure 7 illustrates the microstructure of the cross-section of the tensile specimen. The blue line in Figure 7a delineates the edge of the fracture, while Figure 7b presents an enlarged view of the area enclosed by the green box in Figure 7a. In Figure 7b, several microcracks are observed in the aluminum phase located between the beryllium particles. The width of these cracks is relatively narrow, as indicated by the yellow ellipse markers. However, as illustrated in Figure 7d, the microcracks observed in Be/AlLi exhibit distinct characteristics compared to those in Be/Al. These microcracks predominantly manifest at the interface between the Be and Al phases, highlighted by a red ellipse.

**Figure 8:**
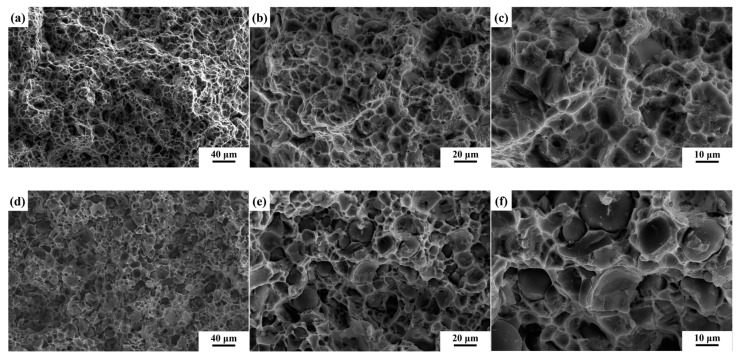
Fracture morphology of composites. (**a**–**c**) Be/Al; (**d**–**f**) Be/AlLi.

For spherical Be power, the interface bonding state can be readily assessed through the analysis of fracture morphology. Figure 8 shows the fracture morphology of the composites. Comparing Figure 8a,b with Figure 8d,e, only a few spherical Be particles can be observed in Be/Al, indicating a good interface bonding state. A greater number of spherical Be particles are observed in the Be/AlLi composite, and significant debonding is evident.

The fracture characteristics of the composites are examined at a higher magnification, as illustrated in Figure 8c,f. In Figure 8c, more dimples can be observed in Be/Al. In the Al phase of Be/AlLi, few dimples can be observed in Figure 8f, but some smooth tearing edges.

The mechanical properties of composites are predominantly influenced by the quality of bonding between the reinforcement and the matrix. Fracture in materials typically occurs at locations exhibiting the lowest strength. It is noteworthy that no significant debonding phenomenon was observed at the Be/Al interface, which suggests that the interfacial bonding strength surpasses that of the matrix material. The mechanical strength of pure aluminum is relatively low; consequently, the initiation point of fracture in Be/Al composites frequently occurs within the aluminum phase. The initial crack widths are typically narrow and challenging to detect [29]. In the Be/AlLi system, a notable occurrence of debonding phenomena has been observed. The observed cracking at the interface can be attributed to the enhanced strength of the aluminum phase; however, there is no significant improvement in interfacial bonding strength. On the other hand, this elucidates the reasons behind the enhancement of Be/AlLi strength. The presence of lithium does not indicate a deterioration in the interfacial bonding between beryllium and aluminum. However, it can be concluded that lithium does not offer any substantial improvement to the interfacial bonding performance of beryllium/aluminum composite materials when compared to the matrix. The improvement of strength in aluminum alloys frequently leads to a decrease in ductility, which can significantly alter the fracture behavior of the alloy. The dimples observed in Figure 8b are not present in Figure 8d. This observation further clarifies the reason behind the decrease in plasticity of Be/AlLi, as illustrated in Figure 6.

## 4. Discussion

### 4.1. Strengthening Mechanism

The strengthening mechanisms of beryllium–aluminum composites can be categorized into the following aspects: (1) dislocation strengthening resulting from work hardening; (2) fine-grain strengthening due to grain refinement; (3) Orowan strengthening induced by sub-micron Be particles; and (4) enhancement of the intrinsic strength of the Al matrix through element addition.

The strengthening effect due to the increase in dislocation density, denoted as ${∆\sigma }_{dis}$, can be determined using Equation (1).

(1) ∆σdis=AGbρ12

In the above equation, the value of A for Al can be taken as 1.25 [30], where G and b represent the shear modulus and Bragg vector of Al, respectively. Utilizing the Dunn formula (2) [31], the dislocation density ρ can be calculated from XRD data.

(2) $$\rho =\frac{L^{2}}{4.35b^{2}}$$

In the above equation, *L* represents the full width at half maximum (FWHM). The calculation results indicate that the dislocation densities for Be/Al and Be/AlLi are 4.33 × 10^13^ m^−2^ and 2.71 × 10^13^ m^−2^, respectively.

The strengthening of fine grains, represented as ${∆\sigma }_{gb}$, can be derived from the classical Hall-Petch Equation (3):

(3) ∆σgb=KD12

K is equal to 0.1 MPa·m^1/2^ [32], and *D* represents the grain size. In this study, the *D* values of Be/Al and Be/AlLi were obtained by processing the Al peak in XRD data (Figure 4), which are 3.5 μm and 3.4 μm, respectively.

When dislocations bypass Be particles, the motion resistance increases. At this point, the strengthening value ${∆\sigma }_{or}$ resulting from Orowan strengthening can be calculated by Equation (4) [33]:

(4) $$\left\{\begin{array}{c} {∆\sigma }_{or}=M\frac{0.4Gb}{\pi \sqrt{1-\upsilon }}\frac{\ln\left(2\bar{r}/b\right)}{\bar{\lambda }}\\ \bar{r}=\frac{D_{P}}{\sqrt{6}}\\ \bar{\lambda }=2\bar{r}\left(\sqrt{\pi /4V_{p}}-1\right) \end{array}\right.$$

$D_{P}$ represents the size of Be particles, which is 17.2 μm. $V_{p}$ denotes the volume fraction of Be particles. The Poisson’s ratio υ is taken to be 0.33. M signifies the orientation factor for Al; a value of 3 is considered reasonable for the materials studied in this study [28]. However, it is generally observed that the contribution of the Orowan strengthening mechanism is minimal. Therefore, it will not be considered in this study.

If we assume that Li enhances the strength of Al alloys through solid solution, then the Al alloy strength $\sigma_{in}$ can be expressed by formula (5) [34].

(5) σin=σ0+∆σss∆σss=∑AiCi23

$\sigma_{0}$ is the Al matrix intrinsic yield stress (25 MPa) [35]. The term ${∆\sigma }_{ss}$ represents the increment in strengthening caused by solute elements. The constant $A_{i}$ is determined by the type of element, with a value of 10.862 for Li [34]. The variable $C_{i}$ denotes the concentration of this specific element.

At this point, the yield strength $\sigma_{my}$ of the aluminum matrix can be determined by Equation (6) [36]:

(6) $$\sigma_{my}=\sqrt{{∆\sigma }_{dis}^{2}+{∆\sigma }_{gb}^{2}+{∆\sigma }_{or}^{2}}+\sigma_{in}$$

According to the modified shear lag model, the yield strength $\sigma_{cy}$ of the composites can be calculated by Formula (7) [37,38]:

(7) $$\sigma_{cy}=\sigma_{my}\left[0.5V_{p}\left(s+2\right)+\left(1-V_{p}\right)\right]$$

In the above equation, s represents the aspect ratio of the Be particles after extrusion. Based on extensive statistical analysis (Figure 2), the value of s is determined to be 4.3.

**Figure 9:**
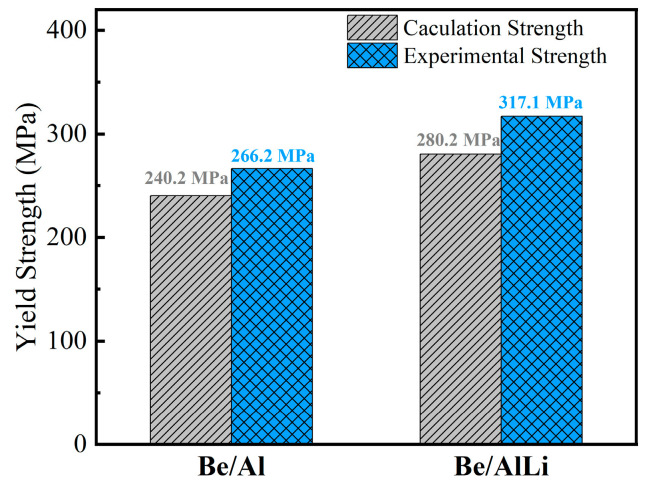
The calculation and experimental yield strength of the composites.

Figure 9 shows the result of the calculation and experimental yield strength of the composites. For Be/Al, the calculated value is 240.2 MPa, while the experimental value is 266.2 MPa. The calculation value is slightly lower than the experimental value, which may be attributed to the uneven distribution of Be particle morphology. The calculation value of Be/AlLi is 280.2 MPa, which is notably lower than the experimental value of 317.1 MPa. It is noteworthy that the modified shear lag model is often more suitable for composites with good interface bonding. Figure 8 indicates that Be/Al has minimal debonding, suggesting good interface adhesion, while Be/AlLi shows significant debonding, indicating poor interface adhesion. Theoretically, the experimental yield strength of Be/AlLi should be significantly lower than the calculation value. However, the results indicate the opposite. This finding suggests that the strength of the AlLi matrix has been severely underestimated.

**Figure 10:**
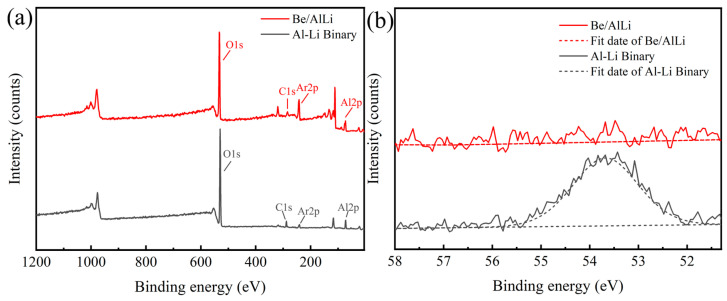
XPS result of Be/AlLi and Al-Li binary. (**a**) Full spectrum; (**b**) high-resolution envelop of the Li 1 s peak.

In the result presented in Figure 9, the contribution of Li to strength is calculated based on the assumption of solid solution strengthening. However, this assumption may be unreasonable. Figure 10 presents the XPS results of Be/AlLi and Al-Li binary. As shown in Figure 10b, the characteristic peak of Li 1 s can be distinctly observed in the Al-Li binary, whereas no corresponding peak is detected in Be/AlLi. Such results may be attributed to both the material’s microstructure and the inherent characteristics of XPS experiments. From the XRD experimental results presented in Figure 4, it can be inferred that Li likely exists in a bulk compound form. The detection depth of XPS is relatively shallow—often less than 10 nm. Any layer of aluminum adhering to these compounds’ surfaces, even extremely thin, would prevent the detection of Li 1 s characteristic peak. These bulk compounds may contribute significantly to enhancing matrix strength in Be/AlLi alloys. Additionally, the XRD data indicate that Be/AlLi and Be/Al exhibit distinct textural characteristics, which may be one of the factors contributing to the observed property differences.

In other studies, it has been found that the Halpin-Tsai formula (8) can effectively predict the elastic modulus E of beryllium–aluminum composites [39].

(8) $$E=E_{m}\left(1+2sqV_{p}\right)/\left(1-qV_{p}\right)$$

where

(9) $$q=\left(E_{p}-E_{m}\right)/\left(E_{p}+2sE_{m}\right)$$

In the above equation, $E_{m}$ represent the elastic moduli of the Al matrix (70 GPa for Al, and 72 GPa for AlLi). $E_{p}$ represent the elastic moduli of the Be powers (290 GPa). After calculation, the elastic moduli of Be/Al and Be/AlLi are 197 GPa and 198 GPa.

### 4.2. Characteristics and Properties of Fractures 

**Figure 11:**
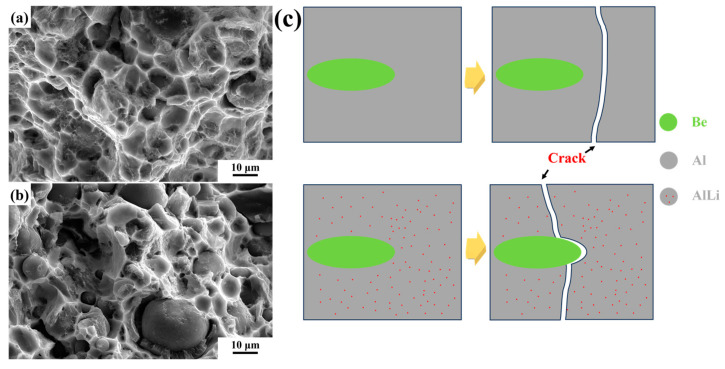
Typical Fracture Morphologies of (**a**) Be/Al, (**b**) Be/AlLi and (**c**) the schematic diagram.

Based on the experimental results presented above, Figure 11 shows the typical fracture morphology of composites along with their schematic diagram. Due to the low strength and high ductility of pure aluminum, microcracks in the Be/Al system initially appear within the aluminum phase. Subsequently, these cracks propagate, merge, and ultimately form a fracture surface. Therefore, the fracture characteristics in the Be/Al predominantly exhibit features associated with the Al phase, characterized by numerous tearing ridges and dimples, as illustrated in Figure 11a. In the Be/AlLi system, the relatively low bonding strength between the Be phase and Al phase leads to a significant occurrence of debonding. As cracks propagate into the Al matrix, material fracture occurs, resulting in the formation of fracture surfaces, as illustrated in Figure 11b.

**Figure 12:**
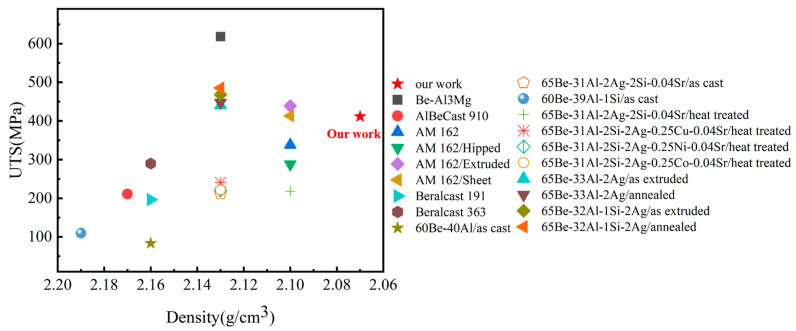
Comparison of the properties of various beryllium–aluminum composites.

The comparison of density and strength for beryllium–aluminum composites with different compositions is presented in Figure 12, and the detailed dates can be found in Table 3. The density of most beryllium–aluminum composites ranges from 2.05 g/cm^3^ to 2.19 g/cm^3^. The beryllium–aluminum composites prepared in this research have a density of 2.07 g/cm^3^, which is generally lower than that of other beryllium–aluminum composites while exhibiting relatively high strength. Alloying is a commonly employed method to enhance the strength of composites. Though elements such as Cu, Mg, Si, and Ag significantly contribute to improving material strength, they often lead to an increase in the density. In comparison, Li can enhance the strength of beryllium–aluminum composites without increasing the density, making it an ideal alloying element.

The casting of traditional Al-Li alloys requires special protection to maintain the chemical state of lithium [46]. In this study, due to the specific nature of the pressure infiltration technology, no special measures were taken to protect the smelting procedure of Al-Li alloys. The lack of protection will lead to significant differences in the as-cast microstructure between Be/Al and Be/AlLi. The casting characteristics of Al-Li alloys determine that the cast microstructure of Be/AlLi tends to exhibit more porosity compared to Be/Al [47,48]; this discrepancy in microstructure can lead to variations in material properties and structure after extrusion. The extrusion process is subjected to triaxial compressive stress, which effectively repairs defects in the as-cast microstructure. As a result, no significant differences in the microstructure were observed in Figure 2. However, the presence of initial defects still considerably impacts the material’s properties. The difference in the interfacial bonding performance between Be/AlLi and Be/Al is significantly influenced by the as-cast microstructure, and XRD results for both composites indicate a severe oxidation condition of Li.

Therefore, in future research, it is essential to investigate the pressure infiltration technology of Al-Li alloys further. On the one hand, it is crucial to utilize argon gas protection during the melting and infiltration stages to reduce Li oxidation reactions. On the other hand, different Li contents in Al-Li alloys exhibit distinct performance characteristics. Therefore, it is essential to examine how different Li concentrations affect the properties and microstructure of beryllium–aluminum composites. By establishing Be/Al-Li composites with gradient Li content, we aim to examine the variations in mechanical properties such as density, elastic modulus, strength, and elongation rate. Additionally, we will explore aspects related to elemental distribution, phase types, and interfacial bonding characteristics within these materials.

## 5. Conclusions

The present study investigates the influence of lithium on the microstructure and mechanical properties of beryllium–aluminum composites by adjusting the matrix composition. The main results are as follows:

(1) The beryllium–aluminum composite materials were prepared using pure aluminum and Al-Li alloy as the matrices. The presence of lithium exacerbated the absorption of oxygen and nitrogen during the infiltration process, resulting in a segregation of generated nitrides and oxides within the matrix.

(2) After adding Li, the yield strength increased from 266.2 MPa to 317.1 MPa, and the tensile strength increased from 348.6 MPa to 411.4 MPa. However, there is a slight decrease in ductility, as it falls from 2.9% to 2.5%. Meanwhile, the density of the composites remains unchanged at 2.07 g/cm^3^. Li is the element that can significantly enhance the properties of beryllium–aluminum composites without increasing the density.

(3) The fracture morphology of the Be/Al without Li predominantly exhibits a ductile Al phase, whereas Be/AlLi reveals a significant occurrence of debonding interfaces. Through an analysis of the reinforcement mechanisms in composite materials, it was found that Li significantly enhances the strength of the aluminum matrix.

## Figures and Tables

**Table 1:** Chemical compositions of the experimental materials (wt.%).

Materials	Al	Li	Be
Be/Al	34.1	-	Bal.
Be/AlLi	33.9	0.2	Bal.

**Table 2:** Tensile properties of composites.

Samples	Yield Strength /MPa	Ultimate Tensile Strength/MPa	Elongation /%
Be/Al	266.2 ± 6.5	348.6 ± 5.7	2.9 ± 0.1
Be/AlLi	317.1 ± 4.5	411.4 ± 3.7	2.5 ± 0.1

**Table 3:** Properties of various beryllium–aluminum composites.

Samples	Density /g cm^−3^	Ultimate Tensile Strength/MPa	Ref
Our work	2.07	411.4	This work
Be-Al_3_Mg	2.13	618	[40]
AlBeCast 910	2.17	211	[41]
AM162	2.10	337.9	[42]
AM162/Hipped	2.10	288	[43]
AM162/Extruded	2.10	439	[43]
AM162/Sheet	2.10	413	[43]
Beralcast 191	2.16	196.5	[44]
Beralcast 363	2.16	289.6	[44]
60Be-40Al/as cast	2.16	83.4	[45]
65Be-31Al-2Ag-2Si-0.04Sr/as cast	2.13	211.0	[45]
60Be-39Al-1Si/as cast	2.19	109.6	[45]
65Be-31Al-2Ag-2Si-0.04Sr/heat treated	2.10	217.9	[45]
65Be-31Al-2Si-2Ag-0.25Cu-0.04Sr/heat treated	2.13	240.6	[45]
65Be-31Al-2Si-2Ag-0.25Ni-0.04Sr/heat treated	2.13	220.0	[45]
65Be-31Al-2Si-2Ag-0.25Co-0.04Sr/heat treated	2.13	221.3	[45]
65Be-33Al-2Ag/as extruded	2.13	440.6	[45]
65Be-33Al-2Ag/annealed	2.13	447.5	[45]
65Be-32Al-1Si-2Ag/as extruded	2.13	468.2	[45]
65Be-32Al-1Si-2Ag/annealed	2.13	485.4	[45]

## Data Availability

The original contributions presented in this study are included in the article. Further inquiries can be directed to the corresponding author.
